# Diel patterns of fin whale 20 Hz acoustic presence in Eastern Antarctic waters

**DOI:** 10.1098/rsos.220499

**Published:** 2023-04-19

**Authors:** Meghan G. Aulich, Brian S. Miller, Flore Samaran, Robert D. McCauley, Benjamin J. Saunders, Christine Erbe

**Affiliations:** ^1^ Centre for Marine Science and Technology, Curtin University, Bentley, WA 6102, Australia; ^2^ Australian Antarctic Division, 203 Channel Highway, Kingston, TAS 7050, Australia; ^3^ Lab-STICC CNRS UMR 6285, ENSTA Bretagne, Brest 29802, France; ^4^ School of Molecular and Life Sciences, Curtin University, Bentley, WA 6102, Australia

**Keywords:** fin whale, *Balaenoptera physalus*, Eastern Antarctica, diel patterns, 20 Hz pulse

## Abstract

This study presents evidence of diel patterns in fin whale (*Balaenoptera physalus*) 20 Hz acoustic presence in Eastern Antarctic waters. Passive acoustic recordings were collected at four sites in Eastern Antarctica from 2013 to 2019. A generalized linear model fitted by a generalized estimating equation was used to test the hypothesis that fin whale 20 Hz acoustic presence shows significant variation between light regimes dawn, day, dusk and night. In the Indian sector of Antarctica, at the Prydz and Southern Kerguelen Plateau sites, fin whale acoustic presence was significantly more common during the night and dawn before declining during the day and dusk periods. A different diel pattern was observed in the Pacific sector, at the Dumont d'Urville site: fin whale acoustic presence was significantly more common during the day than dusk and night periods. No diel pattern was identified at the Casey site. The identified diel patterns in the Indian sector of Eastern Antarctica correlate with previously identified diel patterns of the fin whales' prey. We suggest an indirect association between fin whale acoustic presence and foraging, with the animals more likely to produce the 20 Hz pulse during the night when not foraging and less likely to vocalize when foraging during the day.

## 1. Introduction

Diel patterns of animal vocalizations have been observed throughout marine ecosystems, with species altering the occurrence and production rate of their sounds dependent on the time of day. Diel patterns have been reported in chorus production of many fish species [1] and echolocation clicks of odontocetes [2]. Diel patterns are commonly observed among whale species: the North Pacific right whale (*Eubalaena japonica*), the blue whale (*Balaenoptera musculus*) and the North Atlantic minke whale (*Balaenoptera acutorostrata*) are all reported to produce more calls at night than during the day [3–6]. By contrast, the sei whale (*Balaenoptera borealis*) and sperm whale (*Physeter macrocephalus*) are reported to have greater acoustic presence during the day [7,8].

Diel patterns have also been reported in the occurrence of vocalizations of the fin whale (*Balaenoptera physalus*). The most commonly produced and widely reported vocalization of the fin whale is referred to as the ‘20 Hz’ pulse [9]. This vocalization is characterized by short (approx. 1 s) pulses, which have a frequency range of 42 to 18 Hz [9,10] and are produced in highly stereotyped, repetitive intervals every 7–26 s [9]. The 20 Hz pulse can also be accompanied by the ‘backbeat’ pulse which has a lower frequency range (23 to 13 Hz) and is produced before or after the 20 Hz pulse [10,11]. Other less widely reported vocalizations of the fin whale include the ‘40 Hz’ pulse [12] and higher frequency component [13] which have also been referred to as overtones [14]. The vocalizations of fin whales have been suggested to be associated with behaviour of the animals. Regular sequences of 20 Hz pulses are suggested to be reproductive displays [9] only produced by males [15]. Irregular sequences of 20 Hz pulses are suggested to be associated with social behaviours [16]. Finally, the 40 Hz pulse is suggested to be associated with foraging behaviours [17,18]. As fin whale vocalizations are associated with different behaviours it is reasonable to hypothesize that diel patterns in their acoustic presence are observed among populations.

Throughout the Northern Hemisphere, diel patterns of fin whale acoustic presence have been observed; however, taken together, these patterns vary between and within ocean regions. Populations of fin whales in areas of the Gulf of California produced more 20 Hz pulses during the night than during the day [18]. By contrast, in the Bering Sea, fin whales produced more calls during the day [18]. No diel pattern in fin whale 20 Hz pulse occurrence was observed off Southern California [18]. In the Davis Strait, an anomalous diel pattern of fin whale acoustic presence was observed, with consistent vocalizing throughout the 24 h period, but a clear decline in vocal occurrence at midday [19]. In Canadian Pacific waters, observations are inconsistent, with Pilkington *et al*. [20] identifying a site-specific diel pattern of fin whales calling more during the night than during the day. No diel patterns were identified at other sites in this study or other sites in this region [21]. Further, Burnham [22] observed an apparent, but not statistically significant increase in 20 Hz calls during the night, compared with during the day, at Clayoquot Sound. In Antarctic waters, diel analysis of fin whale acoustic presence is limited and focused on Western Antarctica. Burkhardt *et al*. [23] reported that fin whales at the Western Antarctic Peninsula lacked a diel pattern in 20 Hz pulse presence. By contrast, at the Maude Rise, Shabangu *et al*. [24] found a significant diel pattern with peak 20 Hz pulse rates during the day.

Identification of these diel patterns in acoustic presence of fin whale vocalizations has led to further suggestions of the behavioural ecology of these populations. Night and day diel rhythms in fin whale acoustic presence have led to suggestions of an indirect association with foraging behaviours. The animals are calling less during daytime while foraging for prey and vocalizing more at night when not [19,20].

Using passive acoustic monitoring, the distribution of the fin whale has been identified in Eastern Antarctic waters, with a seasonal acoustic presence of the animals from late austral summer to early winter (January–June) [25]. It is important to note that this study represents acoustic presence of the animals, and a lack of calls does not confirm an absence of fin whales, rather that there are no vocalizing animals present. No studies are available on the diel patterns of acoustic presence of Eastern Antarctic fin whale populations. In this study, we test the hypothesis that acoustic presence of the fin whale 20 Hz pulse follows a diel pattern. Identification of any diel patterns may provide insight into the behavioural ecology and thereby help inform future management of this vulnerable species in Eastern Antarctic waters.

## 2. Materials and methods

**Figure 1.**
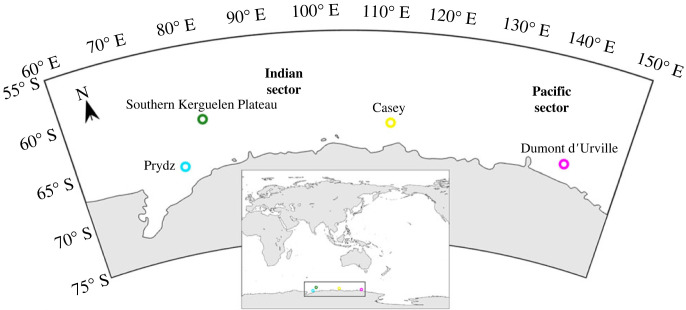
Deployment locations of the MARs used to obtain underwater sounds; equidistant conic projection.

Passive acoustic data were collected using moored acoustic recorders (MARs) of the Australian Antarctic Division. These systems recorded at four locations across Eastern Antarctic waters between the years 2013 and 2019 with varying deployment periods (figure 1, table 1). The MARs had a continuous recording scheme with a sampling frequency of 12 kHz.

**Table 1.** Location, latitude, longitude, recording dates and recorder depth for acoustic recordings at four locations in Antarctic waters from the AAS 4102 Long Term Acoustic Recording Dataset [26].

site and year	latitude (S)	longitude (E)	start date	end date	depth (km)
Prydz					
Prydz 2013	66° 34.484′	77° 39.009′	26 Jan 2013	08 Nov 2013	1.7
Southern Kerguelen Plateau					
Kerguelen 2014	62° 22.806′	81° 47.808′	10 Feb 2014	21 Apr 2015	1.9
Kerguelen 2015	62° 22.818′	81° 47.550′	10 Feb 2015	10 Mar 2016	1.9
Kerguelen 2016	62° 22.176′	81° 41.730′	06 Feb 2016	28 Feb 2017	1.8
Kerguelen 2017	62° 21.606′	81° 42.318′	31 Jan 2017	06 Aug 2017	1.8
Kerguelen 2018	62° 21.894′	81° 42.588′	22 Feb 2018	23 Jan 2019	1.7
Kerguelen 2019	62° 22.620′	81° 47.178′	08 Feb 2019	06 Feb 2020	2.7
Casey					
Casey 2014	63° 47.730′	111° 47.226′	25 Dec 2013	11 Dec 2014	2.7
Casey 2016	63° 48.456′	111° 44.166′	10 Dec 2015	16 Jul 2016	2.7
Casey 2017	63° 48.186′	111° 45.642′	12 Dec 2016	07 Nov 2017	2.7
Casey 2019	63° 48.216′	111° 45.030′	23 Dec 2018	19 Dec 2019	2.7
Dumont d'Urville					
DDU 2018	65° 11.400′	140° 35.898′	05 Feb 2018	05 Oct 2018	2.0
DDU 2019	65° 30.600′	140° 34.896′	31 Dec 2018	10 Dec 2019	2.0

The detection of fin whale pulses at these four Antarctic sites is described in Aulich *et al*. [25], and the detection process is detailed in Aulich *et al*. [27]. Briefly, an automatic detection algorithm was implemented, using the spectrogram cross-correlation method with noise rejection included to remove broadband pulses, followed by a manual checking process to remove samples with false-positive detections and add detections that were missed. This was followed by a time-domain envelope detector and a second manual checking process. The date and time of each fin whale pulse detection was noted.

To test for diel patterns in fin whale 20 Hz acoustic presence, each 24 h period was divided into four light regimes (dawn, day, dusk and night) using the RStudio package *suncalc* [28]. In order to determine these light regimes, the altitude of the sun at each site was calculated at 1 min intervals to determine sunrise, sunset and the start of nautical twilight (i.e. when the sun altitude is between 0 and −12°). Dawn is defined as the hours of and between nautical twilight start and sunrise. Day consists of the hours after sunrise, but before sunset. Dusk consists of the hours from sunset to nautical twilight end. Finally, night is defined as the hours in between the end of and before the start of nautical twilight. The entire hour at the change of sun condition (i.e. at sunrise and sunset) is considered as dawn or dusk. Nautical twilight was used to determine light regimes in order to ensure this study is comparable to the literature on fin whale diel patterns [18,21,23,24].

In order to measure fin whale acoustic presence for our analysis, the hourly presence of detected 20 Hz pulses was noted (present or absent), rather than the number of individual pulses in an hour. These detected fin whale vocalizations were then binned by hour and day of year to create two-dimensional plots of hourly presence and to outline seasonal presence at each site (figure 3). Curves indicating dawn, day, dusk and night were then overlaid to provide an indication of the varying light regime across time at each site. Additionally, fin whale hourly detections were grouped by detection day (days with fin whale acoustic presence), and the mean proportion of presence hours in each regime (dawn, day, dusk and night) was calculated and displayed in bar plots for each site (figure 4).

To test whether any potential patterns in ambient noise could affect diel detections of fin whale 20 Hz pulses, ambient noise was computed in the frequency band of 12–40 Hz for every 1 h wav file across all datasets. Specifically, each wav file was split into a series of successive, non-overlapping 4 s windows. Each 4 s sample was Fourier transformed to give power spectral density, which was integrated from 12 to 40 Hz to compute a band level every 4 s. Over all 4 s samples within each 1 h wave file, the 20th percentile of the ambient noise band level was taken for further analysis. This comparatively low percentile was chosen to exclude samples with nearby and thus high-level fin whale pulses. In the presence of repetitive fin whale calling, this 20th percentile noise value will accurately reflect ambient noise levels. Computed hourly 20th percentile ambient noise levels were binned by light regime and displayed in pirate plots for each site using the RStudio package *YaRrr!* [29] (figure 5).

The effect of light regime on fin whale acoustic presence was statistically analysed using a generalized linear model (GLM) fitted by a generalized estimating equation (GEE) using the RStudio package *geepack* [30–32]. Individual models were run for each site (Prydz, Southern Kerguelen Plateau, Casey and Dumont d'Urville) with light regime as a fixed factor with four levels (dawn, day, dusk and night). The effect of ambient noise level on fin whale acoustic presence was also tested in each model by light regime. Data were conditioned on detection days only (days with 20 Hz acoustic presence in at least one hour). Fin whale acoustic presence data are temporally autocorrelated: the probability of fin whales vocalizing in 1 h is expected to be high if the animals were vocalizing in the previous hour [33]. An autocorrelation function (ACF) plot, supported by a Durbin–Watson test [34] assessed the presence of temporal dependence in the models' residuals (using the RStudio package *car* [35]) and an ‘Ar1’ correlation structure was incorporated into the models.

## 3. Results

**Figure 2.**
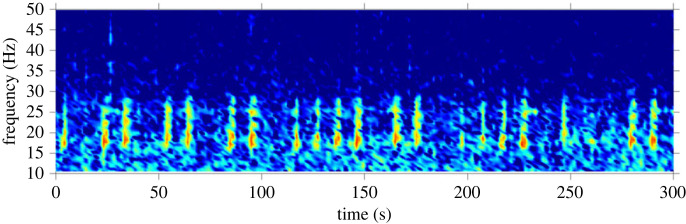
Spectrogram example of a fin whale 20 Hz pulse. Image was taken from Casey (2 May 2019, 20.00). Spectrograms were calculated in 2048-point Hann windows with 0.59 Hz frequency resolution, sampling frequency 1200 Hz.

A total of 4549 h with fin whale acoustic presence was detected across all site-years (table 2). Though quantifying the proportion of different call types and regular or irregular sequences of pulses was beyond the scope of this study, we can report that the regular, stereotyped sequences of 20 Hz pulses were the most commonly detected at every site in this study (figure 2).

**Table 2.** Fin whale detection data for all recording site-years including the first and last detections, the total number of days with acoustic presence and the total number of hours with acoustic presence.

location	year	first detection	last detection	total presence days	total presence hours
Prydz	2013	26 Jan 2013	20 May 2013	38	553
S. Kerguelen Plateau	2014	22 Mar 2014	08 Jun 2014	74	620
	2015	16 Mar 2015	12 May 2015	49	338
	2016	26 Feb 2016	18 May 2016	38	243
	2017	12 Feb 2017	27 May 2017	44	262
	2018	22 Feb 2018	25 May 2018	74	659
	2019	22 Feb 2019	15 Jun 2019	98	854
Casey	2014	28 Feb 2014	31 Mar 2014	8	26
	2016	09 Mar 2016	03 Apr 2016	4	18
	2017	21 Mar 2017	05 May 2017	5	19
	2018	0	0	0	0
	2019	22 Mar 2019	31May 2019	27	258
Dumont d'Urville	2018	7 Feb 2018	10 Jun 2018	59	318
	2019	2 Feb 2019	8 May 2019	57	385

**Figure 3.**
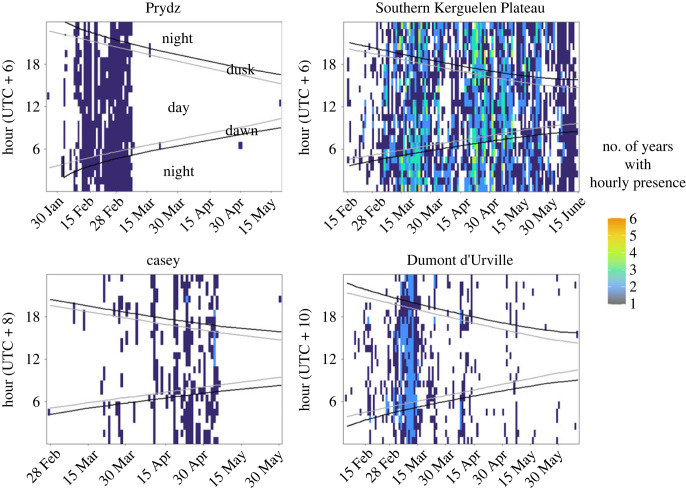
Detections of fin whale 20 Hz vocalizations (presence, absence) as a function of day of year and hour of day for each site. All recording years at Prydz (2013), Southern Kerguelen Plateau (2014–2019), Casey (2014–2019) and Dumont d'Urville (2018–2019) were summed with presence overlapping. Dawn and dusk labels indicate periods of nautical twilight. Note *x*-axes differ for each site, outlining seasonal presence of fin whales at each location.

**Figure 4.**
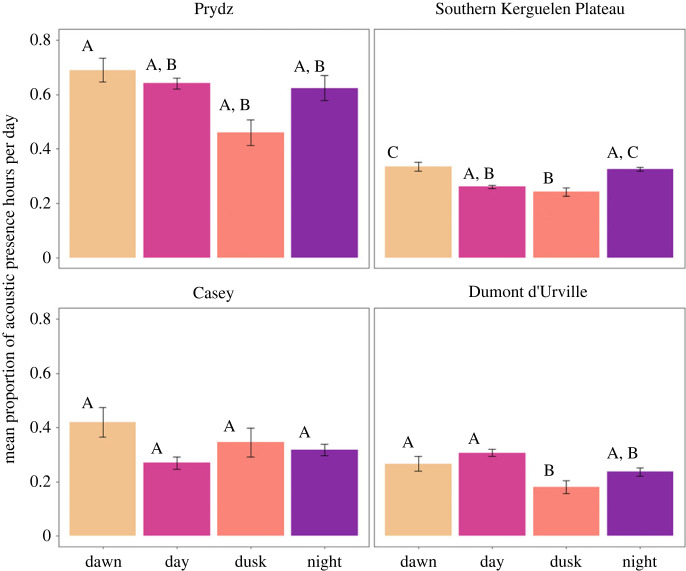
Mean (± s.e.) proportion of 20 Hz acoustic presence hours per detection day in each light regime (dawn, day, dusk and night). Similar letters illustrate statistically similar means (GEE GLM *α* = 0.05).

Previous work recorded a pattern of seasonal acoustic presence of fin whales in Eastern Antarctic waters with the animals present at Prydz from late January to May, at the Southern Kerguelen Plateau from February to June, at Casey from February to May and at Dumont d'Urville from February to June (table 2, figure 3) [25]. The effect of light regime on fin whale acoustic presence differed between sites in Eastern Antarctic waters. In the Indian sector of Antarctica, acoustic presence differed with light regime at both the Prydz and Southern Kerguelen Plateau sites (figure 4). At the Southern Kerguelen Plateau, hours with fin whale acoustic presence were more common during the night and dawn periods before declining during the day and dusk periods (figure 4). At Prydz, a similar effect of light regime on acoustic presence was found, with hours with acoustic presence more common during the night and dawn and reaching a minimum at dusk, although statistical significance only occurred between dawn and dusk periods (figure 4).

In the Pacific sector of Antarctica, at Dumont d'Urville, acoustic presence also differed with light regime; however, the pattern was different to that of the Indian sector sites. Fin whale acoustic presence was more common during the dawn and day than dusk periods (figure 4). At the Casey site, no statistically significant effect of light regime was detected (figure 4), probably due to fewer overall detection hours than at the other sites (table 2).

**Figure 5.**
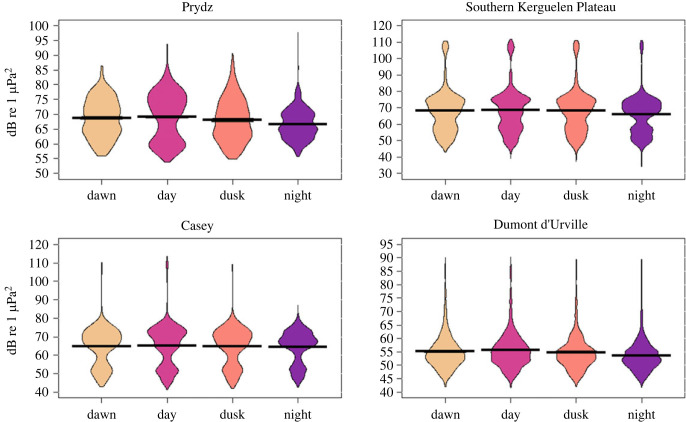
Pirate plots of 20th percentile noise levels within the fin whale call bandwidth in each light regime (dawn, day, dusk and night). Bars represent the mean; beans are the smoothed density curve showing data distribution. Note the different scales for *y*-axes.

The effect of ambient noise level on fin whale acoustic presence differed between sites. At Prydz, noise level was lowest at night and highest during the day; however, hours with acoustic presence were approximately the same during both periods (figures 4 and 5), and no statistically significant effect of noise level was detected (*p* ≥ 0.05). At the Southern Kerguelen Plateau, noise level was lowest during the night; however, hours with acoustic presence were similarly high to those during dawn (figure 4 and 5) and no statistically significant effect of noise level was detected (*p* ≥ 0.05). Ambient noise level at Casey significantly affected hours of fin whale acoustic presence during the day and night (*p* ≤ 0.01 and *p* ≤ 0.01), with hours with acoustic presence more common when noise level was low and acoustic presence less common when noise level was high. However, no diel pattern in ambient noise level was observed at this site (figure 5). Finally, at Dumont d'Urville, ambient noise level significantly affected hours of acoustic presence during the dawn (*p* ≤ 0.01), with hours with acoustic presence less common when noise level was high. Noise level at this site was lowest during the night and highest during the day, which also had the highest acoustic presence hours (figures 4 and 5).

## 4. Discussion

This study identified diel patterns of fin whale 20 Hz acoustic presence at three locations in Eastern Antarctic waters with no diel pattern identified at Casey.

In the Indian sector of Antarctica, fin whales had a greater acoustic presence at night and dawn periods and a lower acoustic presence during day and dusk periods. This nightly increase in call occurrence is consistent with the diel call patterns reported for populations of fin whale in Canadian Pacific waters [20] and a greater acoustic presence at night and dawn in populations of fin whale in the Gulf of California [18].

Aggregation of fin whales in regions of Antarctica is thought to be driven by high-density areas of prey [36,37]. Both the Southern Kerguelen Plateau and Dumont d'Urville locations are likely feeding zones for the animals [25], as they are productive feeding locations for other cetacean species. The main food source of the fin whale in Antarctic waters is the krill species *Euphausia superba* [38], which is distributed throughout Eastern Antarctica, with aggregations identified in waters off Prydz Bay, the Southern Kerguelen Plateau and Dumont d'Urville [39–41]. In Antarctic waters, *Euphausia superba* follow a diel vertical migration pattern (DVM), aggregating at depth during the day and rising in the water column at night [42]. This DVM behaviour is reported to vary seasonally between summer and winter months in regions of Western Antarctica [43,44], however, remains consistent from February to October in the Lazarev Sea [43]. The purpose of this vertical migration is hypothesized to minimize the risk of predation using visual avoidance by occupying deep water during daytime [42,44].

Off California, fin whales exhibit strong diel patterns in their dive behaviour, diving consistently throughout the day and spending prolonged periods in shallow water during the night [45]. This correspondence of daytime dive behaviour with the vertical migration of their food source indicates that the animals are diving to feed during this period and populations of fin whale have been observed to feed during the day [46].

It is possible that the diel patterns in acoustic presence reported here in the Indian sector of Antarctica at Prydz and the Southern Kerguelen Plateau were indirectly associated with feeding behaviour of fin whales. The animals may be less likely to produce the 20 Hz pulse when diving for prey during the day and may be more likely to produce the 20 Hz pulse during the night when feeding is less efficient. Similar suggestions have been made for fin and blue whale populations exhibiting this diel pattern in the Northern Hemisphere [6,20].

While the Dumont d'Urville site largely followed this pattern of greater acoustic presence at night than at dusk, fin whale acoustic presence was most common during the day rather than declining as at the other two sites. This contrast in diel patterns between the Indian and Pacific sectors of Antarctica may reflect differences in prey type and/or availability. This location may not be a consistent feeding zone for the animals, thereby affecting the night and day rhythms. The variability in fin whale diel patterns at Dumont d'Urville may also be a result of variability in the DVM of *Euphausia superba*, where they cease their DVM behaviour in spring and summer months [43]. The DVM behaviour of *E. superba* may occur later in the year at this site, therefore affecting fin whale 20 Hz calling behaviour. The low sample-size and intermittent acoustic presence observed later in the season at Dumont d'Urville may also account for this difference in diel patterns between the Indian and Pacific sectors of Antarctica.

While the acoustic presence of the 20 Hz pulse was significantly affected by light regime at these sites, diel variation was only minimal. Other studies reporting statistically significant variation in fin whale 20 Hz diel presence have observed similar minimal variation between light regimes [18,20]. Our results support the suggestion that the fin whale 20 Hz pulse may be directly associated with reproductive behaviours [9] or social behaviours [16] rather than foraging. Further investigation of the presence and diel occurrence of the 40 Hz call type may help to identify greater diel acoustic patterns of these populations and strengthen our suggestions due to its association with foraging [17].

The identified diel patterns in fin whale 20 Hz acoustic presence across these sites may be influenced by detectability of fin whale pulses by the automatic detection algorithm. Factors affecting the sound propagation environment can impede the detection range of the receivers and thereby total detected fin whale 20 Hz pulses. A range of factors affect propagation, including the bathymetry, water temperature, salinity and ambient noise [47]. Ambient noise in Antarctic waters may be anthropogenic (e.g. ship noise from research vessels), abiotic (e.g. ice cracking, wind) or biotic (marine mammal choruses) [48]. Ambient noise does vary seasonally, particularly in polar waters due to the dampening effect of sea ice coverage [49], and has been reported to vary during day and night periods [50]. A high ambient noise level may decrease the signal-to-noise ratio of fin whale 20 Hz pulses and therefore decrease detectability of the pulse. Ambient noise level analysis in this study identified no consistent effect of noise on fin whale acoustic presence hours across sites. Ambient noise level had no effect on fin whale acoustic presence at Prydz and the Southern Kerguelen Plateau, so we therefore conclude that the observed patterns at these sites were due to diel periodicity in production of 20 Hz pulses. At Dumont d'Urville, while fin whale acoustic presence was affected by noise level at dawn, the observed diel pattern of high acoustic presence during the day was not impacted.

The Casey site was the only location in the Indian sector of Antarctica to not record a diel pattern in fin whale 20 Hz acoustic presence. The lack of a diel pattern at this site was probably due to the long-term pattern of intermittent and inconsistent acoustic presence of fin whales in this region [25]. Fewer animals were vocally present and calling at this location, with Casey suggested as an area of limited use of populations of fin whale in Eastern Antarctica [25]. The increase in fin whale acoustic presence in 2019 was probably responsible for the observed diel variability in acoustic presence. Further acoustic monitoring at this location is required to investigate this increase in fin whale 20 Hz acoustic presence.

Our study highlights variability in diel occurrence and patterns of fin whale 20 Hz acoustic presence across Eastern Antarctic waters. Further, the diel pattern identified in the Indian sector is inconsistent with the diel pattern identified at Maude Rise in Western Antarctic waters [24]. Shabangu *et al*. [24] hypothesized the observed midday peak in 20 Hz call rates may be for the purpose of avoiding vocal competition with vocalizing Antarctic blue whales, which were acoustically abundant at this site. Further acoustic analysis of other species across these four Eastern Antarctic locations may help to ascertain if vocal competition may be a factor contributing to fin whale 20 Hz diel patterns in these regions. Additionally, this variability observed in fin whale 20 Hz diel patterns in Eastern and Western Antarctica outlines the lack of a clear, defined diel pattern of fin whale 20 Hz acoustic presence in Antarctic waters. This indicates that 20 Hz acoustic presence may be population specific throughout Antarctica, thus affirming the need for ongoing and future individual management of populations of this vulnerable species.

## 5. Conclusion

This study has identified the first to date evidence of diel patterns in fin whale 20 Hz acoustic occurrence in Eastern Antarctic waters. The diel pattern of greater acoustic presence during the night and dawn observed in the Indian sector may indicate an indirect association with foraging behaviour: the animals are more likely to call when feeding is less efficient. The Pacific sector site, Dumont d'Urville observed a contrasting pattern with greater acoustic presence during the day and the Casey site observed no diel pattern. This variability in diel patterns observed in regions of Eastern Antarctica, combined with previous Western Antarctic studies, outlines the lack of a consistent diel pattern of fin whale 20 Hz acoustic presence in Antarctic waters.

## Data Availability

The data that support the findings of this study are available from the Australian Antarctic Division (https://data.aad.gov.au/metadata/records/AAS_4102_longTermAcousticRecordings).

## References

[1] Parsons MJ, Salgado-Kent CP, Marley SA, Gavrilov AN, McCauley RD. 2016 Characterizing diversity and variation in fish choruses in Darwin Harbour. ICES J. Mar. Sci. **73**, 2058-2074. (10.1093/icesjms/fsw037)

[2] Osiecka AN, Jones O, Wahlberg M. 2020 The diel pattern in harbour porpoise clicking behaviour is not a response to prey activity. Sci. Rep. **10**, 1-7. (10.1038/s41598-020-71957-0) 32913327 PMC7483526

[3] Munger LM, Wiggins SM, Moore SE, Hildebrand JA. 2008 North Pacific right whale (*Eubalaena japonica*) seasonal and diel calling patterns from long-term acoustic recordings in the southeastern Bering Sea, 2000–2006. Mar. Mamm. Sci. **24**, 795-814.

[4] Risch D, Clark CW, Dugan PJ, Popescu M, Siebert U, Van Parijs SM. 2013 Minke whale acoustic behavior and multi-year seasonal and diel vocalization patterns in Massachusetts Bay, USA. Mar. Ecol. Prog. Ser. **489**, 279-295. (10.3354/meps10426)

[5] Stafford KM, Moore SE, Fox CG. 2005 Diel variation in blue whale calls recorded in the eastern tropical Pacific. Anim. Behav. **69**, 951-958. (10.1016/j.anbehav.2004.06.025)

[6] Wiggins SM, Oleson EM, McDonald MA, Hildebrand JA. 2005 Blue whale (*Balaenoptera musculus*) diel call patterns offshore of Southern California. Aquat. Mamm. **31**, 161. (10.1578/AM.31.2.2005.161)

[7] Baumgartner MF, Fratantoni DM. 2008 Diel periodicity in both sei whale vocalization rates and the vertical migration of their copepod prey observed from ocean gliders. Limnol. Oceanogr. **53**, 2197-2209. (10.4319/lo.2008.53.5_part_2.2197)

[8] Miller BS, Miller EJ. 2018 The seasonal occupancy and diel behaviour of Antarctic sperm whales revealed by acoustic monitoring. Sci. Rep. **8**, 5429. (10.1038/s41598-018-23752-1) 29615756 PMC5882826

[9] Watkins WA, Tyack P, Moore KE, Bird JE. 1987 The 20-Hz signals of finback whales (*Balaenoptera physalus*). J. Acoust. Soc. Am. **82**, 1901-1912. (10.1121/1.395685) 3429729

[10] Thompson PO, Findley LT, Vidal O. 1992 20-Hz pulses and other vocalizations of fin whales, *Balaenoptera physalus*, in the Gulf of California, Mexico. J. Acoust. Soc. Am. **92**, 3051-3057. (10.1121/1.404201) 1474220

[11] Brodie DC, Dunn RA. 2015 Low frequency baleen whale calls detected on ocean-bottom seismometers in the Lau basin, southwest Pacific Ocean. J. Acoust. Soc. Am. **137**, 53-62. (10.1121/1.4904556) 25618038

[12] Miller BS, Calderan S, Leaper R, Miller EJ, Širović A, Stafford KM, Bell E, Double MC. 2021 Source level of Antarctic blue and fin whale sounds recorded on Sonobuoys deployed in the deep-ocean off Antarctica. Front. Mar. Sci. **8**, 792651. (10.3389/fmars.2021.792651)

[13] Miller BS, Balcazar N, Nieukirk S, Leroy EC, Aulich MG, Shabangu FW, Dziak RP, Lee WS, Hong JK. 2021a An open access dataset for developing automated detectors of Antarctic baleen whale sounds and performance evaluation of two commonly used detectors. Sci. Rep. **11**, 1-18. (10.1038/s41598-020-79139-8) 33436710 PMC7804014

[14] Wood M, Širović A. 2022 Characterization of fin whale song off the Western Antarctic Peninsula. PLoS ONE **17**, e0264214. 35271610 10.1371/journal.pone.0264214 PMC8912240

[15] Croll DA, Clark CW, Acevedo A, Tershy B, Flores S, Gedamke J, Urban J. 2002 Only male fin whales sing loud songs. Nature **417**, 809. (10.1038/417809a) 12075339

[16] McDonald MA, Hildebrand JA, Webb SC. 1995 Blue and fin whales observed on a seafloor array in the Northeast Pacific. J. Acoust. Soc. Am. **98**, 712-721. (10.1121/1.413565) 7642810

[17] Romagosa M, Pérez-Jorge S, Cascão I, Mouriño H, Lehodey P, Pereira A, Marques TA, Matias L, Silva MA. 2021 Food talk: 40-Hz fin whale calls are associated with prey biomass. Proc. R. Soc. B **288**, 20211156. (10.1098/rspb.2021.1156) PMC8261222 34229495

[18] Širović A, Williams LN, Kerosky SM, Wiggins SM, Hildebrand JA. 2013 Temporal separation of two fin whale call types across the eastern North Pacific. Mar. Biol. **160**, 47-57. (10.1007/s00227-012-2061-z) 24391281 PMC3873066

[19] Simon M, Stafford KM, Beedholm K, Lee CM, Madsen PT. 2010 Singing behavior of fin whales in the Davis Strait with implications for mating, migration and foraging. J. Acoust. Soc. Am. **128**, 3200-3210. (10.1121/1.3495946) 21110615

[20] Pilkington JF, Stredulinsky E, Abernethy RM, Ford JK. 2018 Patterns of fin whale (Balaenoptera physalus) seasonality and relative distribution in Canadian Pacific waters inferred from passive acoustic monitoring. Ontario, Canada: Canadian Science Advisory Secretariat.

[21] Hendricks B, Keen EM, Shine C, Wray JL, Alidina HM, Picard CR. 2021 Acoustic tracking of fin whales: habitat use and movement patterns within a Canadian Pacific fjord system. J. Acoust. Soc. Am. **149**, 4264-4280. (10.1121/10.0005044) 34241431

[22] Burnham RE. 2019 Fin whale call presence and type used to describe temporal distribution and possible area use of clayoquot sound. Northwest Sci. **93**, 66-74. (10.3955/046.093.0106)

[23] Burkhardt E et al. 2021 Seasonal and diel cycles of fin whale acoustic occurrence near Elephant Island, Antarctica. R. Soc. Open Sci. **8**, 201142. (10.1098/rsos.201142) 34084537 PMC8150045

[24] Shabangu FW, Andrew RK, Yemane D, Findlay KP. 2020 Acoustic seasonality, behaviour and detection ranges of Antarctic blue and fin whales under different sea ice conditions off Antarctica. Endanger. Species Res. **43**, 21-37. (10.3354/esr01050)

[25] Aulich MG, McCauley RD, Miller BS, Samaran F, Giorli G, Saunders BJ, Erbe C. 2022 Seasonal distribution of the fin whale (*Balaenoptera physalus*) in Antarctic and Australian waters based on passive acoustics. Front. Mar. Sci. **9**, 864153. (10.3389/fmars.2022.864153)

[26] Miller BS, Milnes M, Whiteside S. 2021 Long-term underwater acoustic recordings 2013–2019, Ver. 4. Kingston, Australia: Australian Antarctic Data Centre. (10.26179/h7xa-y729)

[27] Aulich MG, McCauley RD, Saunders BJ, Parsons MJG. 2019 Fin whale (*Balaenoptera physalus*) migration in Australian waters using passive acoustic monitoring. Sci. Rep. **9**, 8840. (10.1038/s41598-019-45321-w) 31222147 PMC6586899

[28] Thieurmel B, Elmarhraoui A. 2019 Suncalc: compute sun position, sunlight phases, moon position and lunar phase. R Package version 50. See https://cran.r-project.org/web/packages/suncalc/suncalc.pdf (accessed 3 March 2022).

[29] Phillips ND. 2017 *YaRrr! The Pirate's Guide to R* [Online]. See https://www.psychologicalscience.org/observer/yarrr-the-pirates-guide-to-r (accessed 15 March 2023).

[30] Højsgaard S, Halekoh U, Yan J. 2006 The R Package geepack for generalized estimating equations. J. Stat. Softw. **15**, 1-11.

[31] Yan J. 2002 Yet another package for generalized estimating equations. R-News **2/3**, 12-14.

[32] Yan J, Fine JP. 2004 Estimating equations for association structures. Stat. Med. **23**, 859-880. (10.1002/sim.1650) 15027075

[33] Salgado Kent C, Marques TA, Harris D. 2022 Fundamental data analysis tools and concepts for bioacoustical research. In Exploring animal behavior through sound: volume 1: methods (eds C. Erbe, J. A. Thomas). Cham, Switzerland: Springer International Publishing.

[34] Durbin J, Watson GS. 1971 Testing for serial correlation in least squares regression. III. Biometrika **58**, 1-19. 14801065

[35] Fox J, Weisberg S. 2019 An R companion to applied regression. Thousand Oaks, CA: Sage.

[36] Reid K, Brierley AS, Nevitt GA. 2000 An initial examination of relationships between the distribution of whales and Antarctic krill *Euphausia superba* at South Georgia. J. Cetacean Res. Manage. **2**, 143-150.

[37] Santora JA, Schroeder ID, Loeb VJ. 2014 Spatial assessment of fin whale hotspots and their association with krill within an important Antarctic feeding and fishing ground. Mar. Biol. **161**, 2293-2305. (10.1007/s00227-014-2506-7)

[38] Mizroch SA, Rice DW, Breiwick JM. 1984 The fin whale, *Balaenoptera physalus*. Mar. Fish. Rev. **46**, 20-24.

[39] Jarvis T, Kelly N, Kawaguchi S, van Wijk E, Nicol S. 2010 Acoustic characterisation of the broad-scale distribution and abundance of Antarctic krill (*Euphausia superba*) off East Antarctica (30-80°E) in January-March 2006. Deep Sea Res. (II Top. Stud. Oceanogr.) **57**, 916-933. (10.1016/j.dsr2.2008.06.013)

[40] Matsuno K, Wallis JR, Kawaguchi S, Bestley S, Swadling KM. 2020 Zooplankton community structure and dominant copepod population structure on the southern Kerguelen Plateau during summer 2016. Deep Sea Res. Part II **174**, 104788. (10.1016/j.dsr2.2020.104788)

[41] Nicol S, Kitchener J, King R, Hosie G, William K. 2000 Population structure and condition of Antarctic krill (*Euphausia superba*) off East Antarctica (80–150 E) during the Austral summer of 1995/1996. Deep Sea Res. (II Top. Stud. Oceanogr.) **47**, 2489-2517. (10.1016/S0967-0645(00)00033-3)

[42] Zhou M, Dorland RD. 2004 Aggregation and vertical migration behavior of *Euphausia superba*. Deep Sea Res. Part II **51**, 2119-2137. (10.1016/j.dsr2.2004.07.009)

[43] Cisewski B, Strass VH, Rhein M, Krägefsky S. 2010 Seasonal variation of diel vertical migration of zooplankton from ADCP backscatter time series data in the Lazarev Sea, Antarctica. Deep Sea Res. Part I **57**, 78-94. (10.1016/j.dsr.2009.10.005)

[44] Taki K, Hayashi T, Naganobu M. 2005 Characteristics of seasonal variation in diurnal vertical migration and aggregation of Antarctic krill (*Euphausia superba*) in the Scotia Sea, using Japanese fishery data. CCAMLR Sci. **12**, 163-172.

[45] Keen EM, Falcone EA, Andrews RD, Schorr GS. 2019 Diel dive behavior of fin whales (*Balaenoptera physalus*) in the Southern California Bight. Aquat. Mamm. **45**, 233-243.

[46] Tershy BR. 1992 Body size, diet, habitat use, and social behavior of Balaenoptera whales in the Gulf of California. J. Mammal. **73**, 477-486. (10.2307/1382013)

[47] Erbe C, Peel D, Smith JN, Schoeman RP. 2021 Marine acoustic zones of Australia. J. Mar. Sci. Eng. **9**, 340. (10.3390/jmse9030340)

[48] Erbe C et al. 2019 Managing the effects of noise from ship traffic, seismic surveying and construction on marine mammals in Antarctica. Front. Mar. Sci. **6**, 647. (10.3389/fmars.2019.00647)

[49] Insley SJ, Halliday WD, de Jong T. 2017 Seasonal patterns in ocean ambient noise near Sachs Harbour, Northwest Territories. Arctic **70**, 239-248. (10.14430/arctic4662)

[50] Baumann-Pickering S, Roch MA, Wiggins SM, Schnitzler HU, Hildebrand JA. 2015 Acoustic behavior of melon-headed whales varies on a diel cycle. Behav. Ecol. Sociobiol. **69**, 1553-1563. (10.1007/s00265-015-1967-0) 26300583 PMC4534505

[51] Meghan GA, Robert DM, Brian SM, Flore S, Giacomo G, Benjamin JS, Christine E. 2022 Seasonal Distribution of the Fin Whale (Balaenoptera physalus) in Antarctic and Australian Waters Based on Passive Acoustics. Frontiers In Marine Science **9**, 864153. (10.3389/fmars.2022.864153)

